# Combinatorial Study of Phase Composition, Microstructure and Mechanical Behavior of Co-Cr-Fe-Ni Nanocrystalline Film Processed by Multiple-Beam-Sputtering Physical Vapor Deposition

**DOI:** 10.3390/ma15062319

**Published:** 2022-03-21

**Authors:** Péter Nagy, Nadia Rohbeck, Remo N. Widmer, Zoltán Hegedűs, Johann Michler, László Pethö, János L. Lábár, Jenő Gubicza

**Affiliations:** 1 Department of Materials Physics, Eötvös Loránd University, H-1518 Budapest, Hungary; nagyp@student.elte.hu; 2 Laboratory for Mechanics of Materials and Nanostructures, EMPA Swiss Federal Laboratories for Materials Science and Technology, Feuerwerkerstrasse 39, CH-3602 Thun, Switzerland; nadiarohbeck@me.com (N.R.); remo.widmer@empa.ch (R.N.W.); johann.michler@empa.ch (J.M.); laszlo.petho@empa.ch (L.P.); 3 Deutsches Elektronen-Synchrotron DESY, Notkestr. 85, 22607 Hamburg, Germany; zoltan.hegedues@desy.de; 4 Institute for Technical Physics and Materials Science, Centre for Energy Research, H-1053 Budapest, Hungary; labar.janos@ek-cer.hu

**Keywords:** multiple-beam-sputtering physical vapor deposition, compositional complex alloy, microstructure, hardness, elastic modulus

## Abstract

A combinatorial Co-Cr-Fe-Ni compositional complex alloy (CCA) thin film disk with a thickness of 1 µm and a diameter of 10 cm was processed by multiple-beam-sputtering physical vapor deposition (PVD) using four pure metal sources. The chemical composition of the four constituent elements varied between 4 and 64 at.% in the film, depending on the distance from the four PVD sources. The crystal structure, the crystallite size, the density of lattice defects (e.g., dislocations and twin faults) and the crystallographic texture were studied as a function of the chemical composition. It was found that in a wide range of elemental concentrations a face-centered cubic (fcc) structure with {111} crystallographic texture formed during PVD. Considering the equilibrium phase diagrams, it can be concluded that mostly the phase composition of the PVD layer is far from the equilibrium. Body-centered cubic (bcc) and hexagonal-close packed (hcp) structures formed only in the parts of the film close to Co-Fe and Co-Cr sources, respectively. A nanocrystalline microstructure with the grain size of 10–20 nm was developed in the whole layer, irrespective of the chemical composition. Transmission electron microscopy indicated a columnar growth of the film during PVD. The density of as-grown dislocations and twin faults was very high, as obtained by synchrotron X-ray diffraction peak profile analysis. The nanohardness and the elastic modulus were determined by indentation for the different chemical compositions on the combinatorial PVD film. This study is the continuation of a former research published recently in Nagy et al., Materials 14 (2021) 3357. In the previous work, only the fcc part of the sample was investigated. In the present paper, the study was extended to the bcc, hcp and multiphase regions.

## 1. Introduction

Compositionally complex alloys (CCAs) or multi-principal element alloys (MPEAs) contain three or more principal elements with similar compositions, i.e., the elements cannot be categorized as solute or solvent atoms unlike in the conventional alloys [1,2]. For many compositions, CCAs are single-phase materials due to the stabilization effect of the enhanced configuration entropy. If the configuration entropy is higher than 1.61*R*, where *R* is the universal gas constant $\left(8.314\frac{J}{K*mol}\right)$, the material is called as high-entropy alloy (HEA) [3]. This criterion for the configuration entropy is usually achieved if the material contains at least five principal elements with equal or nearly equal compositions [3]. The emerging interest in HEAs is based on their improved properties compared to the conventional alloys, such as the combination of high strength and good ductility [4]. There are compositions which can preserve their high strength even at elevated temperatures; these are the refractory HEAs [5]. It has transpired that CCAs with three principal elements may exhibit a better performance than HEAs with more (e.g., five) constituents. For instance, CoCrNi medium entropy alloy (MEA) had a higher ultimate tensile strength, ductility, and toughness than for CoCrFeMnNi HEA [6]. The better mechanical performance of CoCrNi MEA was attributed to its lower stacking fault energy (SFE) which yielded a higher degree of dislocation dissociation and twinning even in the early stage of deformation. The consequently higher strain hardening caused an improved ductility and a higher ultimate tensile strength for CoCrNi MEA as compared to CoCrFeMnNi HEA [6]. Therefore, not only HEAs but also MEAs are worth developing and investigating in the CCA family of materials.

CCAs can be manufactured not only in bulk form by casting [7] or powder metallurgy [8] techniques but also as layers and coatings [9]. The reasons for the manufacturing of thin film CCAs are their improved mechanical strength [10,11] and corrosion resistance [12] compared to the behavior of conventional metals and alloy layers. Different sputtering methods have been applied in the literature for the production of CCA coatings [13,14,15,16,17]. These techniques often produce microstructures which are far from the equilibrium phase composition [14]. It has been shown that the conditions of sputtering can influence the chemical composition and the microstructure of the sample [16]. In addition, the microstructure and the phase composition can be tailored by varying the concentrations of the principal chemical elements in order to achieve the best combination of the functional and mechanical properties of CCA films [18]. The most powerful way of the investigation of the effect of chemical composition on crystal structure and microstructure (grain size, texture, lattice defect density) for a given set of elements is the production and study of a combinatorial sample. There have been successful attempts for production and study of combinatorial CCA thin-film specimens in the literature [17,19,20,21]. Recent studies proved the capability of co-sputtering of metals for producing combinatorial thin films [22,23,24,25]. Due to the unique multiple independent sources, these layers exhibited well-defined compositional gradients on the surface. Therefore, the relationship between the concentrations of the constituent elements, the phase composition and the performance of the material can be studied on a single sample.

**Figure 1:**
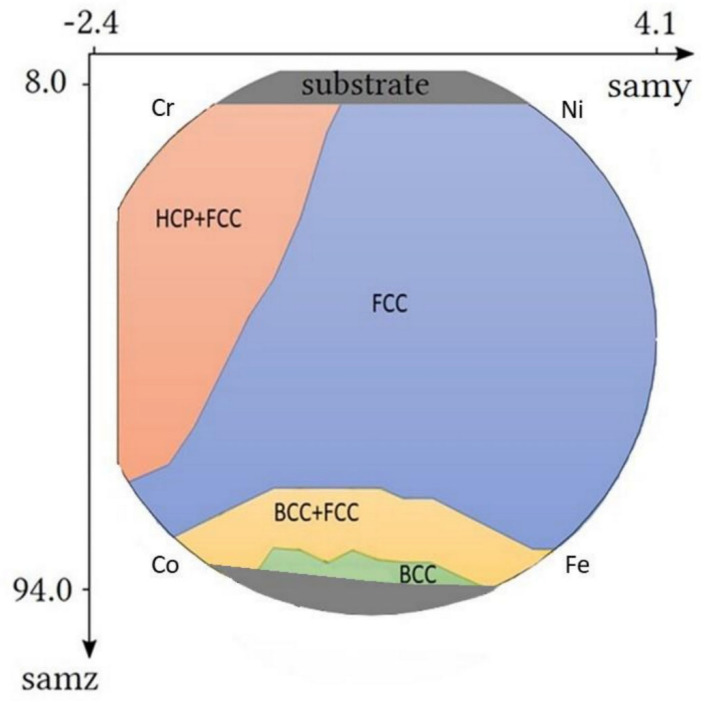
The phase map of the Co-Cr-Fe-Ni CCA sample.

The present study is a continuation of a recently published paper in which a combinatorial Co-Cr-Fe-Ni CCA thin film processed by multiple-beam sputtering (MBS) was investigated [21]. CoCrFeNi composition can be regarded as the base alloy of many HEAs, such as the most-studied Cantor alloy (CoCrFeMnNi) [10,18,20,26]. The chemical composition of the four constituent elements in the Co-Cr-Fe-Ni CCA film varied between 4 and 64 at.%, depending on the distance from the four PVD sources. In the former paper, the crystallite size, the dislocation density and the twin fault probability were determined by X-ray line profile analysis (XLPA) as a function of the position on the surface of the combinatorial thin film, i.e., the microstructure versus the chemical composition was mapped. On the other hand, this investigation has been performed only on that area of the film where the material was a single face-centered cubic (fcc) phase. There was only one exception where the main phase was a hexagonal close-packed (hcp) structure beside the minor fcc phase. In the present study, the investigation of the microstructure is extended to those areas where the main phase is body-centered cubic (bcc) or hcp, or multiphase structures are developed. Figure 1 shows that these parts of the Co-Cr-Fe-Ni CCA thin film can be found at the edges of the disk shape sample close to Co, Fe and Cr sources. In addition, the hardness and the elastic modulus are mapped using nanoindentation technique. One of the aims of the present study is to reveal whether equilibrium phases are formed in the MBS-processed thin film for the different chemical compositions. Further motivation is to find the correlation between chemical composition, phase content, microstructure and mechanical performance in the Co-Cr-Fe-Ni system. As the CCA thin films can be potentially used as hard coatings, our goal is to find the composition for the highest hardness.

## 2. Materials and Methods

### 2.1. Processing of the Combinatorial Film and Selection of the Studied Locations

**Figure 2:**
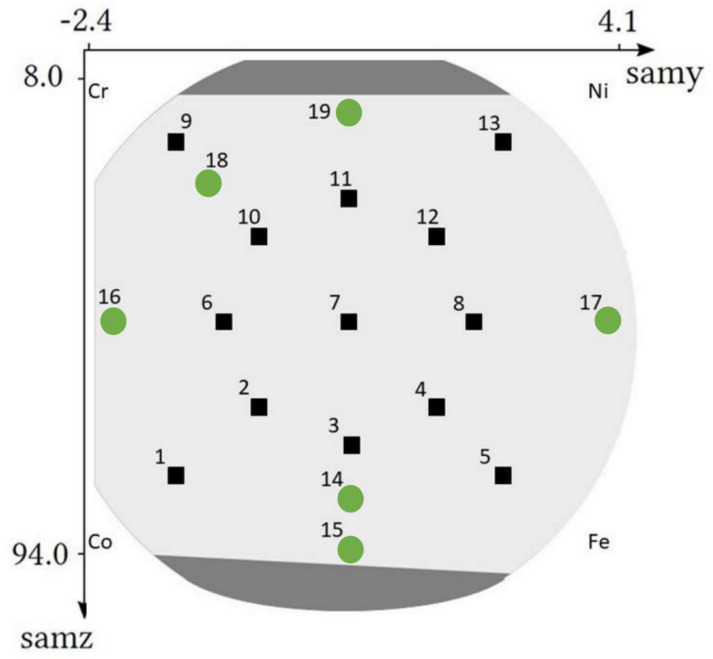
The points on the Co-Cr-Fe-Ni thin film surface investigated in this study (numbered from 1 to 19). The approximate positions of Cr, Ni, Co and Fe sputtering sources are also shown. The masked parts of the Si substrate, indicated by dark grey color, were not covered by CCA film. The areas indicate due to the masking effect of the sample holder. The points investigated in our previous work [21] are marked by black squares while the additional locations studied in this paper are indicated by green circles.

The combinatorial sample was manufactured by a novel PVD method known as multiple beam sputtering (manufacturer: Polygon Physics, Fontaine, France). This technique uses multiple independent ion sources; therefore, premanufactured CCA targets are not required for the preparation of CCA thin film samples. The power feeding of the plasma sources in the sputtering system was 3 W, and the vacuum was 2 × 10^−7^ mbar. The spacing between the wafer and the target was 15 cm. The deposition lasted for 30 h. More details on the applied sputtering technique are given in Ref. [21]. The spherical geometry of the source ring produces well defined element gradients on the surface of the sample; thus, the investigation of a vast chemical combination of the target materials is possible. Our previous work investigated 13 points on the sample, where the dominant phase was fcc [21]. These points are marked as black squares in Figure 2. In this work, we extend the investigation with 6 additional points marked as green circles (see Figure 2). These points were selected in the peripheral parts of the disk sample where, besides the fcc phase, bcc and hcp structures also formed. Namely, between the Co and Fe sources, bcc structure was observed. Here, two points were selected: one in the single bcc phase area (point No. 15) and another in the two-phase fcc + bcc region (point No. 14) in order to investigate the transition between fcc and bcc. The point in position No. 18 was selected between two previously investigated points Nos. 9 and 10 in which the structures were almost pure hcp and fcc, respectively. Three additional points were also studied to cover the remaining main areas, namely, the near edge position between Co and Cr (position No. 16), Ni and Fe (point No. 17), and Cr and Ni (point No. 19).

### 2.2. Study of the Chemical Composition

The concentrations of the four constituent elements as a function of the position on the thin film sample were measured by an X-ray fluorescence (XRF) spectrometer (type: XDV-SDD, manufacturer: Helmut Fischer Holding AG, Hünenberg, Switzerland). Tungsten X-ray radiation and a Peltier-cooled Silicon Drift Detector (SDD) were used in these experiments. The chemical composition was investigated in 360 spots. The measurements were taken in a pattern consisting of 8 concentric circles with different radii. The difference between the radii of the neighboring circles was 5 mm. Each circle contained 40 measuring points, i.e., the polar angle difference between the neighboring points in a circle was 9°. The diameter of each examined point was 1 mm.

### 2.3. Investigation of the Microstructure and the Crystallographic Texture

The microstructure of the sample was investigated by synchrotron X-ray diffraction (XRD) at the Deutsches Elektronen-Synchrotron (DESY), in Hamburg, Germany. The beam energy was 44 keV which corresponds to the X-ray wavelength of λ = 0.028178 nm. The horizontal and vertical step size for the mapping was set to 268 µm and 2000 µm, respectively, giving a horizontal step size on the sample of 4040 µm due to the low angle between the incident beam and the sample surface (3.84°). The diffraction images were recorded in the range of 8–94 mm vertically and from −2.4 mm to 4.1 mm (excluding the end position) horizontally in the cartesian coordinate system attached to the sample, resulting in 44 × 25 = 1100 positions. Although only 19 points were used for a detailed microstructure investigation, the phase map shown in Figure 1 was constructed from all the diffraction patterns obtained in 1100 points on the sample surface.

The Nelson–Riley [27] method was used to obtain the lattice constants from the XRD peak positions. The microstructural parameters at each investigated point were obtained by X-ray line profile analysis (XLPA) [28]. The evaluation of the diffractograms were carried out by the Convolutional Multiple Whole Profile (CMWP) fitting procedure [29]. Using this analysis, the microstructure was characterized by the following quantities: the area-weighted mean crystallite size (<x>_area_) and the dislocation density (ρ). For the points having fcc structure, the twin fault probability (β) was also determined. This quantity represents the fraction of the faulted {111} planes in fcc materials.

The multiphase microstructure of the film was also examined by a scanning transmission electron microscope (STEM). Focused ion beam (FIB) technique using Ga ions was used in a Scios 2 Dual Beam microscope (Thermo Fisher Scientific, Waltham, MA, USA) to prepare a lamella from the samples for (S)TEM. The TEM foil preparation has been described in Ref. [21]. A Titan Themis G2 200 transmission electron microscope (Thermo Fisher Scientific, Waltham, MA, USA) was used for taking the TEM bright-field (BF) and high-angle annular dark-field (HAADF) pictures. The details of recording TEM images and diffraction patterns have been given in Ref. [21]. Automatic processing of the diffraction patterns and creation of the phase map from them was conducted by the DiffMap program, developed by one of the authors [30].

The crystallographic texture was characterized using pole figure measurements. The pole figures were taken by a Smartlab X-ray diffractometer (manufacturer: Rigaku, Tokyo, Japan) using parallel-beam optics and CuKα radiation (wavelength: 0.15418 nm). Three of the newly added points (Nos. 14, 16 and 18) were excluded, since in these points the diffraction peaks of the different phases were strongly overlapped, which makes the measurement of the pole figures impossible.

### 2.4. Nanoindentation

The mechanical properties of the CCA sample at the points of interest were investigated by nanoindentation using an Ubi-1 Nanoindenter equipped with a diamond Berkovich tip (manufacturer: Hysitron, Eden Prairie, MN, USA). To ensure the characterization of the thin film without the effect of the substrate, the maximal load was chosen to be 2 mN. Nine measurements were performed at each location. More details about the hardness measurement can be found in Ref. [21]. The elastic modulus and the hardness were calculated from the indentation curves using the method of Oliver and Pharr [31].

## 3. Results

### 3.1. Chemical and Phase Composition in the Studied Locations of the Film

**Figure 3:**
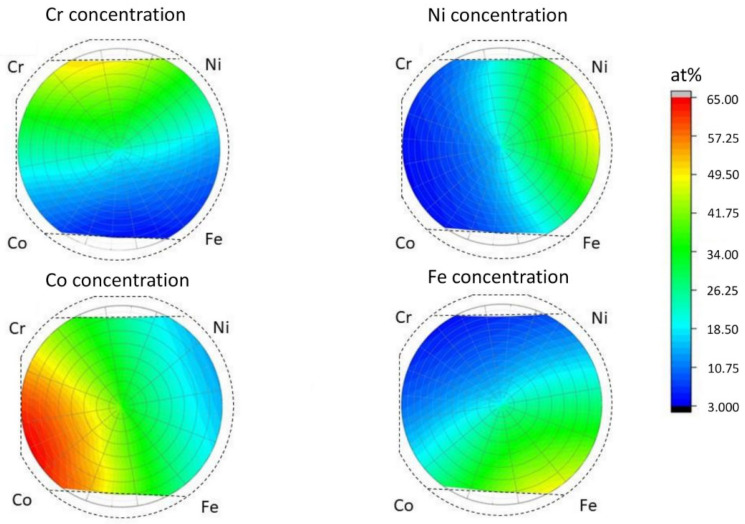
The XRF maps of the 4 initial elements. The dotted line represents the contour of the wafer.

The chemical composition of the thin film in the whole disk was obtained by XRF. The concentration maps for the four constituent elements are shown in Figure 3. In the case of Cr, Fe, and Ni, the concentration varied in the range of 5–48 at.%. In the case of Co, the concentrations were between 10 and 61 at.%. The different concentration range for Co can be explained by the higher sputtering rate of this element. The accumulated sputtering rate of Co, Cr, Fe, and Ni were 14.7, 10, 8.8 and 8.8 nm/h respectively. The concentrations of the four elements in the 19 points of interest are listed in Table 1.

**Figure 4:**
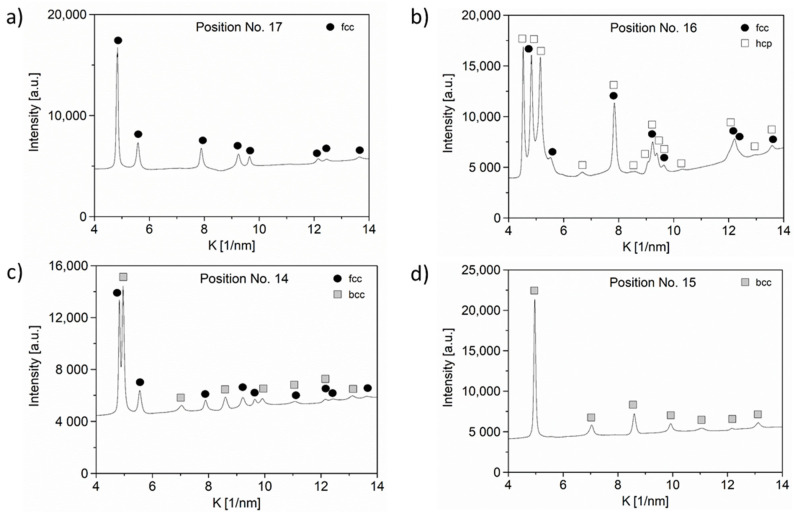
X-ray diffractograms for points Nos. 17 (**a**), 16 (**b**), 14 (**c**) and 15 (**d**). K = 2sinθ/λ, where θ is the Bragg angle, and λ is the wavelength of X-rays.

The phase composition obtained for the points of interest by XRD is shown in Table 1. The phase map obtained for the whole disk is presented in Figure 1. For the majority of the sample, i.e., for a wide range of concentrations, the alloy has an fcc structure. This is valid for the middle of the disk and the edge parts between the Ni and Fe sources (point No. 17) as well as the Cr and Ni sources (location No. 19). As an example, Figure 4a shows the diffraction pattern for point No. 17. Between the Cr and Co sources (e.g., in point No. 16), fcc and hcp phases coexist, as revealed by the diffractogram in Figure 4b. In point No. 14, both bcc and fcc structures were observed (see Figure 4c). From point No. 14 into the direction of the edge of the sample, a single-phase bcc structure was detected, as illustrated by the diffraction pattern taken for point No. 15 (see Figure 4d). The points corresponding to the phase transitions from fcc to bcc or hcp will be further investigated by TEM.

The lattice parameters for fcc, hcp, and bcc phases were obtained from the XRD patterns and listed in Table 1. The lattice constants in position No. 16 were not determined, due to the heavily overlapping peaks of the hcp and fcc phases.

### 3.2. Characterization of the Microstructure by XLPA and TEM

**Figure 5:**
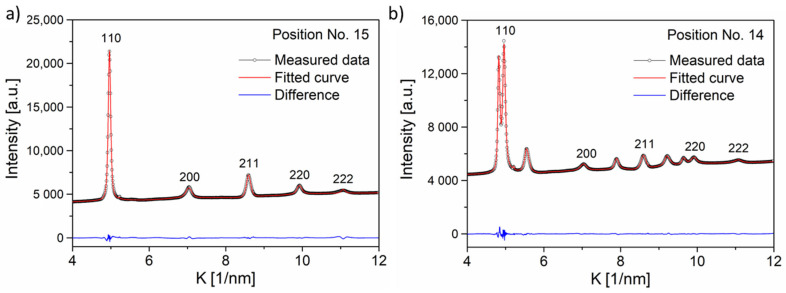
Evaluation of the first five XRD peaks of the bcc phase at points Nos. 15 (**a**) and 14 (**b**) applying CMWP fitting procedure. The peaks of the fcc phase were put into the background during the CMWP fitting.

The characterization of microstructure was carried out by XLPA. Figure 5 shows the CMWP fitting for positions No. 15 and 14 where the peaks of the major bcc phase were evaluated. The microstructural parameters such as the crystalline size, dislocation density, and twin-fault probability obtained by CWMP fitting are listed in Table 2. The twin-fault probability was determined only for the fcc phase.

**Figure 6:**
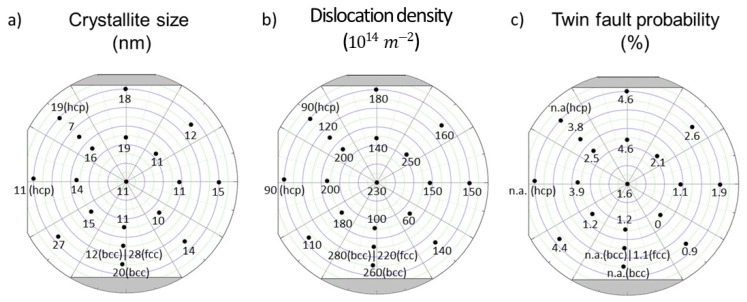
The parameters of the microstructure determined by XLPA for different locations on the Co-Cr-Ni-Fe film.

The crystallite size, the dislocation density, and the twin-fault probability as a function of location on the CCA sample are shown in Figure 6. The crystallite size varied between 7 and 28 nm. Both the largest and lowest crystalline sizes were obtained from locations with dual phase structures. The dislocation density changes between 60 and 280 × 10^14^ m^−2^. The twin-fault probability values for points with fcc phase were between 0.9 and 4.6%, except for point No. 4, where twin-faults were not detected.

**Figure 7:**
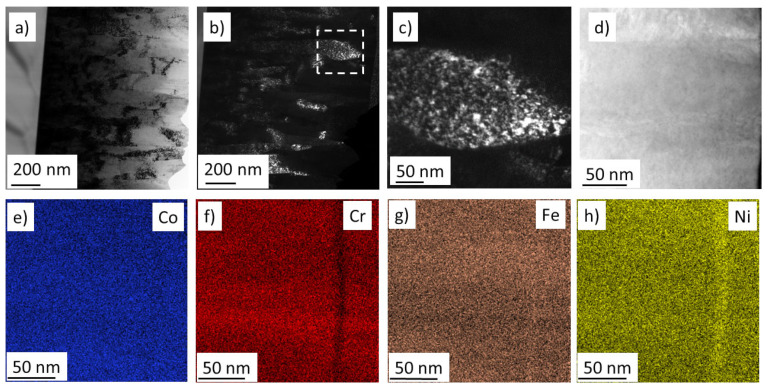
(**a**) Bright-field and (**b**) dark-field TEM images taken on the cross-section of the film at point No. 3. (**c**) A magnified region from figure (**b**) indicated by the dashed square, showing the fragmentation of a column into grains. (**d**) HAADF image and (**e**–**h**) the corresponding element maps for the four constituents.

**Figure 8:**
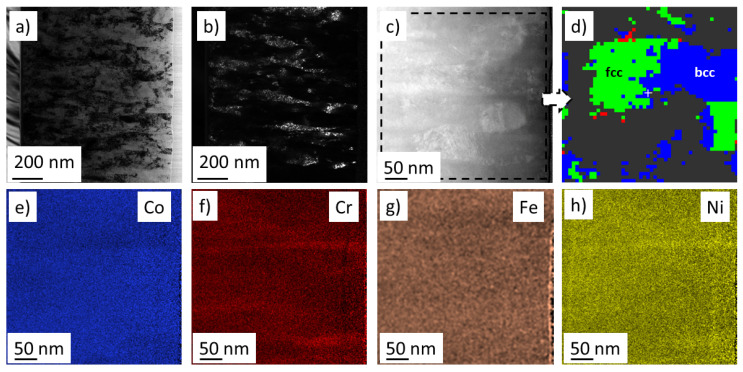
(**a**) Bright-field and (**b**) dark-field TEM images on the cross-section of the film at point No. 14 where dual phase fcc/bcc structure formed. (**c**) HAADF image and (**d**) the corresponding phase map obtained by diffraction in STEM. The green and blue regions represent the fcc and bcc phases, respectively, while in the grey areas the phase identification was uncertain. (**e**–**h**) Element maps for the four constituents in the region corresponding to figure (**c**,**d**).

**Figure 9:**
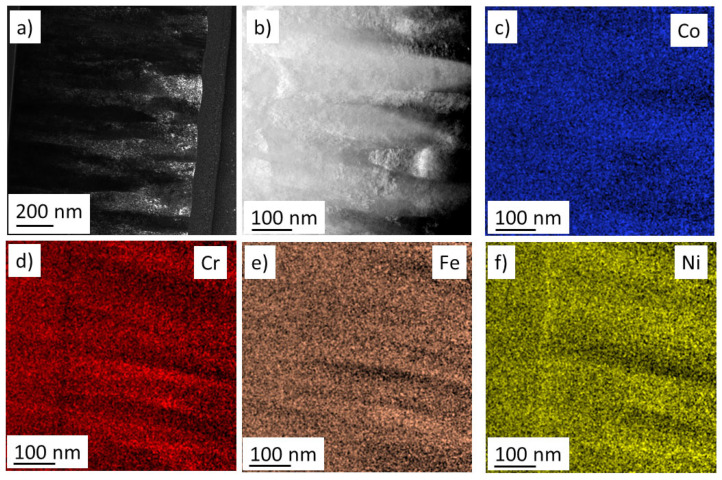
(**a**) Dark-field TEM image of the film cross-section at point No. 15. (**b**) HAADF image and (**c**–**f**) the corresponding element maps for the four constituents.

In our previous studies, TEM investigations were carried out on the cross-section of the disk at the equimolar point No. 7 [17] and the hcp/fcc dual phase region No. 9 [21]. In the present work, the TEM investigation focuses on the areas where there is a transition between fcc and bcc phases. Therefore, three neighbor points were investigated: one with a single-phase fcc structure (No. 3), a second one with a dual phase fcc/bcc structure (No. 14), and a third one with a single-phase bcc structure (No. 15). The cross-sectional microstructure for point No. 3 can be seen in Figure 7a. Similarly to the previously investigated points, locations Nos. 3, 14 and 15 also have a columnar structure (for points Nos. 14 and 15, see Figure 8a and Figure 9a, respectively). The columns have a thickness of about 100–200 nm. The columns are fragmented into smaller grains, as revealed by the DF images in Figure 8b,c for point No. 3, where the reflecting grains have a bright contrast. Similar grains with bright contrast can be seen for the regions Nos. 14 and 15 in Figure 8b and Figure 9a, respectively. The grain size values obtained by TEM are about 10 nm, which are in a reasonable agreement with the crystallite size determined by XLPA as shown in Table 2.

Element maps were also taken for positions Nos. 3, 14 and 15 in STEM using energy dispersive X-ray spectroscopy (EDS). Figure 7d, Figure 8c and Figure 9b show the HAADF images corresponding to the areas studied by EDS for points Nos. 3, 14 and 15, respectively. The EDS element maps of the four constituents for locations Nos. 3, 14 and 15 are presented in Figure 7, Figure 8 and Figure 9, respectively. Only small chemical fluctuations can be observed on the element maps. For instance, in Figure 8 and Figure 9 wavy-like concentration variation appears in which there are Cr and Fe rich regions. In the Cr rich regions, the Cr concentration is 1 at.% higher than the average, while the concentration of Fe is lower with the same amount. In the Fe rich zones, a reverse change of the Cr and Fe contents was observed. The concentrations of Co and Ni did not change significantly between these regions. For the multiphase region No. 14, Figure 8d shows the neighboring fcc and bcc structures identified by diffraction in STEM. The size of these volumes is about 100–200 nm, and thus they should be polycrystalline regions, since the DF images suggested a grain size of about 10 nm. Figure 8 also indicates that there is no significant difference between the chemical compositions of the fcc and bcc structures.

**Figure 10:**
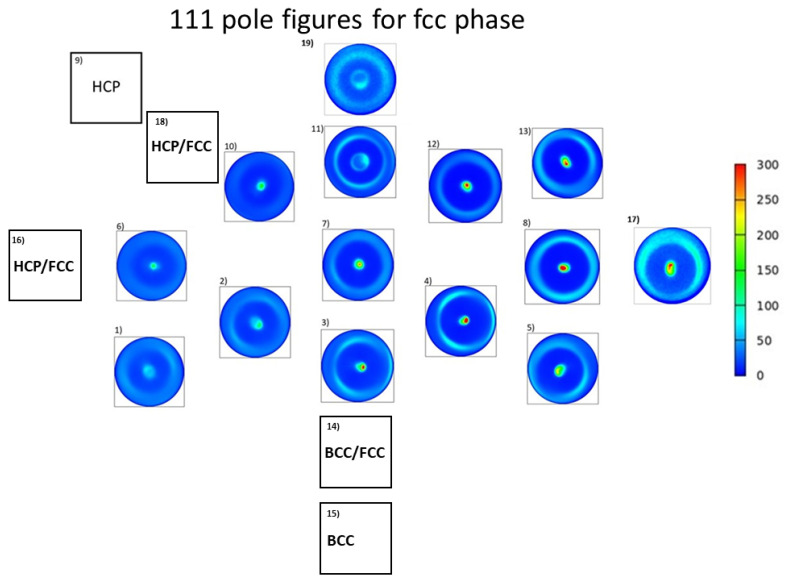
The 111 pole figures determined by XRD for the fcc phase in the different points of the Co-Cr-Ni-Fe film. The blank squares indicate locations where the structure was different from fcc.

**Figure 11:**
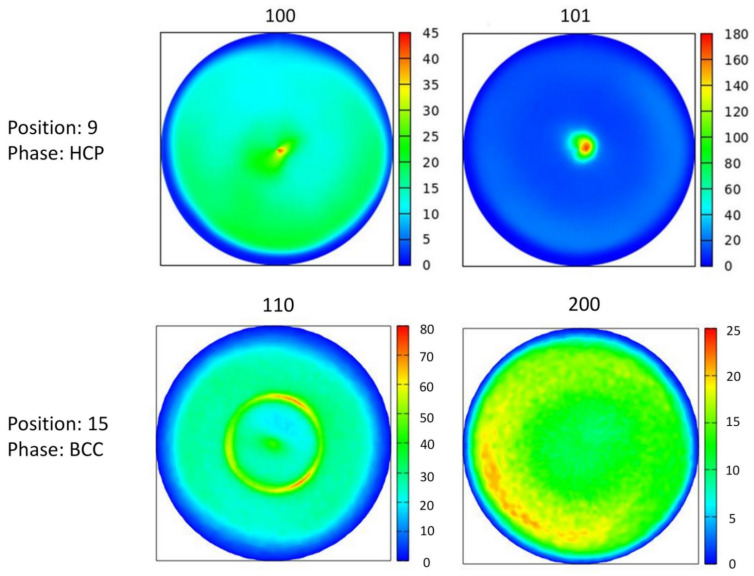
The 100 and 101 pole figures measured by XRD for the major hcp phase at location No. 9. The 110 and 200 XRD pole figures characterizing the texture for the bcc phase at point No. 15.

For fourteen positions where the structure was fcc, the crystallographic texture was characterized by 111 pole figures, as shown in Figure 10. For each point of interest, 111 fiber texture was detected. For position No. 9, where the major phase was hcp, the texture was characterized by 100 and 101 pole figures, indicating the existence of both 100 and 101 components in the texture, as shown in Figure 11. In the case of position No. 15, where a single-phase bcc structure was observed, the texture was characterized by the 110 and 200 pole figures. Two texture components were identified at this location: a stronger 111 and a weaker 110 component (see Figure 11). In points Nos. 14, 16 and 18 with multiphase structures, the texture could not be measured due to the heavily overlapping diffraction peaks of the different phases.

### 3.3. Hardness and Elastic Modulus as Determined by Nanoindentation

**Figure 12:**
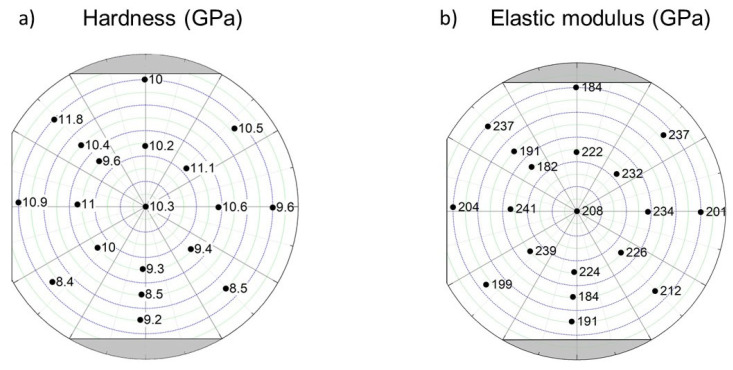
The distribution of the hardness (**a**) and the elastic modulus (**b**) for the Co-Cr-Ni-Fe thin film as determined by nanoindentation.

The mechanical behavior of the Co-Cr-Fe-Ni CCA film was investigated by nanoindentation. The obtained values of the hardness and the elastic modulus are listed in Table 3. The hardness varies between 8.4 and 11.8 GPa, while the elastic modulus values were in the range of 182–241 GPa. The hardness and elastic modulus distribution in the film surface were plotted in Figure 12. Systematic changes in the mechanical behavior were not observed.

## 4. Discussion

### 4.1. Comparison between the Experimentally Determined Phase Composition and the Equilibrium Phase Diagrams

The present synchrotron XRD analysis revealed the influence of the chemical composition on the crystalline phase evolution in Co-Cr-Fe-Ni system processed in the form of a 1 µm-thick film using a multibeam sputtering technique. Figure 1 and Table 1 show that in the majority of the PVD disk an fcc phase formed, irrespective of the element concentrations. This behavior is in accordance with a previous study which predicts the development of an equilibrium fcc structure in the Co-Cr-Fe-Ni system when the concentrations of all the four constituent elements varied between 19 and 40 at.% [32]. In our case, this condition is valid strictly only for point No. 7. However, there are other two locations (Nos. 8 and 12) where this requirement for the concentration is fulfilled for three constituents while the amount of the fourth element is only slightly lower (16 at.%) than the required lower limit of 19 at.%. For these two points, we can also accept that the observed single-phase fcc structure is in thermodynamic equilibrium. Table 4 compares the experimentally determined phase composition and the theoretically predicted equilibrium one for the nineteen studied points.

In locations Nos. 3, 4 and 5, which can be found below the area bordered by points Nos. 7, 8 and 12 (see Figure 2), the experimentally determined structure is also a single-phase fcc (see Table 1 and Table 4). In these points, at least for one constituent the concentration is about 10 at.% or lower. Since a full Co-Cr-Fe-Ni phase diagram at RT cannot be found in the literature (it was determined only for 800 °C or higher temperatures [33]), the equilibrium phase compositions at these points are not available. Therefore, an estimation of the equilibrium phase content was performed on the basis of room temperature ternary phase diagrams for the locations where one constituent has a relatively low concentration (about 10 at.% or lower). In these cases, the concentration of the latter constituent was distributed among the three major elements in accordance with their fractions. For instance, in point No. 3 the chemical composition is approximated as Co_44_Fe_39_Ni_17_, and the Co-Fe-Ni ternary phase diagram reveals that this composition forms a mixture of fcc and bcc structures [34,35], i.e., the phase content of the present material is not in thermodynamic equilibrium at location No. 3. For point Nos. 4 and 5, the compositions were close to Co_40_Fe_32_Ni_28_ and Co_49_Fe_31_Ni_20_, respectively. The Co-Fe-Ni ternary phase diagram [34,35] suggests an fcc + bcc dual phase structure also for these compositions; therefore, in points Nos. 4 and 5, the phase content did not reach its equilibrium state during the film deposition.

At the right side of the fcc area, in point No. 17, the main three elements form the following approximate ternary composition: Ni_54_Fe_30_Cr_16_. According to the Cr-Fe-Ni phase diagram, the equilibrium phase composition corresponds to a single-phase fcc structure, in accordance with the experimental observation (see Table 4) [36]. On the top of the experimentally observed fcc area (see Figure 1), in locations Nos. 11 and 19, the approximate ternary compositions were very close, namely, Cr_45_Co_30_Ni_25_ and Cr_47_Co_32_Ni_21_, respectively (see Table 1). Cr-Co-Ni ternary phase diagram at RT was not found in the literature. The lowest temperature was 800 °C for which Cr-Co-Ni phase diagram was available [37,38,39]. For the compositions corresponding to locations Nos. 11 and 19, fcc γ and tetragonal σ phases coexist at 800 °C, and between 800 and 1200 °C, the area of the fcc phase in the phase diagram is reduced with decreasing the temperature. Therefore, it is expected that at RT the concentration range for the γ phase is even lower than at 800 °C, i.e., the experimentally observed single-phase fcc structure is most probably not in thermodynamic equilibrium. Nevertheless, since Cr-Co-Ni ternary phase diagram is not available at RT, equilibrium phase composition was not listed for points Nos. 11 and 19 in Table 4.

At the bottom part of the Co-Cr-Fe-Ni film, between Co and Fe PVD sources, a bcc structure was formed. In locations Nos. 14 and 15, the Cr content is very low (see Table 1), and the chemical composition can be approximated as Co_43_Fe_41_Ni_16_ and Co_40_Fe_44_Ni_16_, respectively. Therefore, the Co-Fe-Ni ternary phase diagram was used for the determination of the equilibrium phases. The phase diagram suggests that for this composition the equilibrium structure at RT is a mixture of fcc and bcc phases [35], which is in accordance with the experimentally determined phase composition for point No. 14 (see Table 1 and Table 4). If we are going from point No. 3 to location No. 15 via point No. 14, the structure transforms from fcc to bcc while the only considerable change in the chemical composition is the decrease in the Cr concentration from 11 to 6 at.% at the expense of the Fe content (see Table 1). It is well known that Cr addition up to 10 at.% reduces the temperature of bcc to fcc phase transition in Fe, i.e., for lower Cr content, the bcc structure is more stable [40]. This is in accordance with the increased bcc fraction along the path marked by points Nos. 3, 14 and 15. However, in location No. 15 the full bcc structure is not in equilibrium, since the Co-Fe-Ni ternary phase diagram suggests a dual phase fcc+bcc structure [35].

The last remaining location at the right side of the disk is point No. 13, where Cr and Ni were the two major elements with similar fractions, and there are additional Co and Fe having concentrations of 12 and 10 at.%, respectively. Neglecting iron, the approximate ternary composition is Ni_50_Cr_38_Co_12_. Since, Cr-Co-Ni ternary phase diagram is not available at RT, the equilibrium phase composition cannot be estimated. However, using the two major constituents only, the Cr-Ni binary phase diagram suggests that for equimolar Cr-Ni alloy, the main phase is the orthorhombic CrNi_2_ [38]. In addition, a secondary bcc Cr phase also forms. Therefore, most probably, the experimentally observed single-phase fcc structure is not in thermodynamic equilibrium. Nevertheless, since Cr-Co-Ni ternary phase diagram is not available at RT, equilibrium phase composition was not listed for points No. 13 in Table 4.

At the bottom of the left side of the Co-Cr-Fe-Ni disk, for locations Nos. 1 and 2 the approximate three constituent compositions are Co_64_Fe_26_Cr_10_ and Co_56_Fe_27_Cr_17_, respectively. Although Co-Fe-Cr ternary phase diagram was published 90 years ago [41], the two component sections of this diagram (e.g., Co-Fe) are not in accordance with the corresponding binary phase diagrams. Since an updated ternary diagram was not found in the literature, the equilibrium phase composition could not be determined for locations Nos. 1 and 2. The same was valid for point Nos. 6 and 16.

At the upper left corner of the thin film disk, the experimentally determined structure changes from fcc to hcp when departing from the center towards the periphery along points Nos. 10, 18 and 9 (see Figure 2 and Table 1). In location No. 10, the approximate three constituent composition is Co_47_Cr_39_Ni_14_. As mentioned above, room temperature ternary Co-Cr-Ni phase diagram is not accessible in the literature. The available phase diagram at 800 °C suggests that this composition corresponds to a phase mixture consisting of fcc, hcp and tetragonal Σ-phases [37,38,39]. When the temperature decreases down to 800 °C, the fcc area in the phase diagram is reduced; therefore, most probably, the experimentally observed full fcc structure in point No. 10 is not in equilibrium. The same is valid for location No. 18. Nevertheless, for points Nos. 10 and 18, equilibrium phases are not presented in Table 4, since ternary Co-Cr-Ni phase diagram is not available at RT.

For location No. 9, both Fe and Ni have concentrations significantly lower than 10 at.% and the major constituents Cr and Co have nearly equal fractions. Therefore, the composition can be approximated as CoCr. The Co-Cr binary phase diagram predicts that Co and Cr cannot be dissolved in each other at RT, therefore the coexistence of pure hcp Co and bcc Cr phases with similar fractions is suggested in thermodynamic equilibrium [34]. Thus, the experimentally observed phase composition with a major hcp phase in point No. 9 does not represent the equilibrium state. Comparing the measured and the equilibrium phase compositions for the different locations in Table 4, it can be concluded that thermodynamic equilibrium was achieved surely only for the area bounded by points Nos. 7, 8, 17 and 12, i.e., in the middle-right side of the Co-Cr-Fe-Ni thin film.

### 4.2. Variation of the Microstructure and the Mechanical Performance as a Function of the Position on the Combinatorial Film Surface

The crystallite size determined by XLPA varies between 7 and 28 nm in the studied 19 points of the Co-Cr-Fe-Ni thin film. These values are in accordance with the grain size values (dimension of the bright spots in the DF-TEM images) as shown in Table 2. This means that the columns growing perpendicular to the film surface consist of nanocrystalline grains. The dislocation density changes in the range of 60–280 × 10^14^ m^−2^, while the twin-fault probability in the fcc phase varies between 0.9 and 4.6%, except for point No. 4, where twin-faults were not detected. Correlation between the chemical composition and the lattice defect densities was not revealed. At the same time, it is worth noting that the lattice defect density values observed in the present CCA layer are similar as those for other conventional alloy thin films studied formerly in the literature. For instance, for Ni–6 at.% Mo layer processed by electrodeposition, the dislocation density and the twin fault probability were found to be about 180 × 10^14^ m^−2^ and 6%, respectively [42]. Such high values can also be obtained in pure electrodeposited metal films if the electrolyte bath contains high concentration of organic additives. For example, saccharin or cysteine additives resulted in dislocation densities of about 200–600 × 10^14^ m^−2^ and twin fault probability of 3–4% in Ni layers [43,44]. This indicates that the high lattice defect density observed for the present sample is not a unique feature of CCA thin films. It should be noted, however, that the average grain and crystallite sizes for the present CCA film (about 10–15 nm, see Table 2) is smaller than that for the conventional Ni and Ni-Mo films (about 30 nm).

For the equimolar Co-Cr-Fe-Ni composition, the microstructure developed in the thin film can be compared with the bulk nanostructure obtained for the same composition by severe plastic deformation (SPD). In CoCrFeNi CCA processed by 10 turns of high pressure torsion (HPT), the crystallite size was refined to about 30 nm [45]. In the present thin film, at the equimolar point the crystallite size value was much lower (see location No. 7 in Table 2). Moreover, the dislocation density and the twin fault probability in the HPT-processed CoCrFeNi sample were ~150 × 10^14^ m^−2^ and ~2.6%, respectively, i.e., they have similar order of magnitude as in the equimolar location of the present thin film. This comparison suggests that the density of grown-in lattice defects in nanocrystalline CCA coatings can reach or even exceed the defect density achieved by SPD-processing on similar compositions. This is also valid for non-CCA materials [46,47].

The nanohardness and the elastic modulus for the studied nineteen locations as a function of the position on the film surface are shown in Figure 12. The hardness varied between 8.4 and 11.8 GPa. The lowest hardness values (about 8–9 GPa) were obtained at the bottom of the combinatorial sample where the structure is fcc and/or bcc. On the other hand, the highest hardness was determined for point 9 where the main phase is hcp. For hcp structures, the number of easy-slip systems is much lower than for fcc or bcc phases, which can yield a more difficult plastic deformation, i.e., a higher hardness. In addition, the hardness is also influenced by the grain size and the defect density. It should be noted, however, that the effect of grain size is hard to describe, since the size of the grains (about 10–20 nm) falls into the transient range between the Hall–Petch and inverse Hall–Petch behavior; therefore, it is dubious whether the decrease in the grain size causes hardening or softening, or has no significant effect on the hardness (corresponding to the plateau on the Hall–Petch plot) [46]. Therefore, the correlation between the hardness and the microstructure cannot be examined quantitatively. The elastic modulus values determined by nanoindentation for the different locations on the surface of the Co-Cr-Fe-Ni thin film were in the range of 184–241 GPa. Similar to the hardness, a relationship between the chemical composition and the Young’s modulus was not found. The phase composition, the concentrations of the constituents and the crystallographic texture surely influence the elastic modulus. However, it should be noted that for the majority of the disk with fcc structure, the texture is very similar (see Figure 10); therefore, it has only a negligible contribution to the differences observed in the mechanical behavior at the different points on the disk surface. On the other hand, the change of the surface roughness could also yield variation in the mechanical performance probed by nanoindentation, since our coating was not atomically flat. In addition, a small amount of porosity may exist in the PVD film, which can also influence the hardness and the elastic modulus. The variation of porosity in the film may contribute to the difference between the mechanical behavior observed in the different locations. Nevertheless, it can be concluded that the highest hardness (~11.8 GPa) in the combinatorial Co-Cr-Fe-Ni CCA thin film was achieved for composition 42% Co–45% Cr–5% Fe–8% Ni (at.%) where the major phase was hcp. It is worth noting that this hardness was exceptionally high compared to the values determined for the Co-Cr-Ni-Fe system in the literature (between 2.3–8.5 GPa) [11,48,49,50,51]. For instance, for magnetron-sputtered, nano-twinned fcc CoCrFeNi thin film with equimolar composition the hardness was obtained as ~8.5 GPa. The much higher maximum hardness in the presently studied layer (~11.8 GPa) can be attributed to the harder hcp structure as discussed above.

Regarding the practical application of Co-Cr-Ni-Fe CCA thin films, they have not only advanced mechanical properties, such as high strength and toughness, but also can be used as nuclear protective coatings due to their excellent irradiation tolerance [52]. In the latter case, the delayed damage accumulation is attributed to high defect recombination. Furthermore, similarly to stainless steels, CCAs demonstrate superior corrosion resistance, due to the presence of some elements which facilitate the foundation of passive protective layers. In the current case, the formation of a Cr-rich oxide surface produces a barrier against the attack of aggressive ions from the environment.

## 5. Conclusions

The microstructure and the mechanical behavior as a function of the chemical composition were studied on a combinatorial Co-Cr-Ni-Fe CCA thin film produced by multiple-beam-sputtering PVD. One important advantage of the MBS method is that due to the geometrical arrangement of the sputtering sources, this processing way allows for coating substrates with a relatively low surface topography. The following conclusions are obtained:

1. For most of the thin film, a single-phase fcc structure was formed. Close to the periphery of the disk sample between Fe-Co and Co-Cr sources, the main phases were bcc and hcp, respectively. Comparison between the experimentally determined phases and the equilibrium phase diagrams revealed that the phase composition was not in thermodynamic equilibrium for the majority of the combinatorial sample. Equilibrium single-phase fcc region was developed in the middle of the disk where the four constituents have nearly equal fractions.
2. The thin film consists of columns growing perpendicular to the layer surface. These columns are fragmented into spherical nanograins with the size of about 10–20 nm, irrespective of the chemical composition. These grains correspond to the crystallites whose size was determined by XLPA. Very high dislocation density (60–280 × 10^14^ m^−2^) and twin fault probability (0.9–4.6%) were also observed by XLPA for all studied locations. Correlation between the chemical composition and the lattice defect density was not revealed.
3. In all fcc points, a strong 111 fiber texture was observed. For the hcp phase, both 100 and 101 texture components co-exist. Between Fe and Co PVD sources, where a single-phase bcc structure was identified, the texture was characterized by a stronger 111 and a weaker 110 components.
4. Although, strict correlation between the mechanical performance and the composition could not be determined, it was revealed that the highest hardness (~11.8 GPa) was achieved for the composition 42% Co–45% Cr–5% Fe–8% Ni (at.%). In this point, the major phase was hcp which has a lower number of easy-slip systems compared to fcc or bcc structures, resulting in a higher hardness.

In the present sputtering methodology, coatings with only four chemical constituents were manufactured. Our future research plan is to increase the number of sputtered elements from four to six in the processing of combinatorial PVD layers. In this way, we will obtain HEA coatings instead of the presently investigated MEA layers. Then, the correlation between the chemical composition, phase content, microstructure and mechanical behavior will be studied, similar to this work. In addition, the thermal stability of the phase composition and the microstructure will be investigated using controlled annealing processes.

## Figures and Tables

**Table 1:** Chemical composition, phase content and lattice constants for the studied points. The coordinates y and z of these locations are also shown. In the case of dual phase positions, the minor phase is indicated by brackets.

No. of Position	y	z	Composition from XRF [at.%]				Phase Composition	Lattice Constant [nm]
			Co	Cr	Fe	Ni		
1	−1.596	82	61	10	24	5	fcc	0.360 ± 0.001
2	−0.524	70	51	15	24	10	fcc	0.360 ± 0.001
3	0.548	76	39	11	35	15	fcc	0.360 ± 0.001
4	1.352	70	24	13	35	28	fcc	0.360 ± 0.001
5	2.156	84	19	6	46	29	fcc	0.359 ± 0.001
6	−1.06	56	52	23	16	9	fcc	0.360 ± 0.001
7	0.548	56	31	23	23	23	fcc	0.361 ± 0.001
8	2.156	56	16	19	27	38	fcc	0.360 ± 0.001
9	−1.596	26	42	45	5	8	hcp + (fcc)	a = 0.258 ± 0.001 (hcp)
								c = 0.417 ± 0.001 (hcp)
10	−0.524	42	42	35	11	12	fcc	0.362 ± 0.001
11	0.548	36	27	40	11	22	fcc	0.362 ± 0.001
12	1.352	44	23	31	16	30	fcc	0.361 ± 0.001
13	2.424	30	10	34	12	44	fcc	0.361 ± 0.001
14	0.548	82	39	8	38	15	fcc + bcc	0.359 ± 0.001 (fcc)
								0.285 ± 0.001 (bcc)
15	0.548	90	38	6	41	15	bcc	0.285 ± 0.001
16	−2.132	56	61	21	13	5	hcp + fcc	a = 0.254 ± 0.001 (hcp)
								c = 0.413 ± 0.001 (hcp)
17	2.96	56	10	14	27	49	fcc	0.359 ± 0.001
18	−0.792	30	40	39	9	12	fcc + (hcp)	0.363 ± 0.001 (fcc)
								n. a.
19	0.548	12	29	43	9	19	fcc	0.365 ± 0.001

**Table 2:** The microstructural parameters as determined by XLPA using the CMWP fitting method (<x>_area_: area-weighted mean crystallite size; ρ: dislocation density; and β: twin-fault probability). In the first column, beside the numbers of the investigated point the evaluated phases are also indicated which is important in the cases of the multiphase structures (for the phase composition, see Table 1). The grain size values determined for some locations by TEM are also shown.

No. of Position	<x>_area_ [nm]	ρ [10^14^ m^−2^]	β [%]	d_TEM_ [nm]
1-fcc	27 ± 4	110 ± 10	4.4 ± 0.5	
2-fcc	15 ± 2	180 ± 20	1.2 ± 0.1	
3-fcc	11 ± 2	100 ± 10	1.2 ± 0.1	14 ± 2
4-fcc	10 ± 2	60 ± 10	0 ± 0.1	
5-fcc	14 ± 2	140 ± 20	0.9 ± 0.1	
6-fcc	14 ± 2	200 ± 20	3.9 ± 0.4	
7-fcc	11 ± 2	230 ± 30	1.6 ± 0.2	
8-fcc	11 ± 2	150 ± 20	1.1 ± 0.1	
9-hcp	19 ± 3	90 ± 10	n.a.	
10-fcc	16 ± 2	200 ± 20	2.5 ± 0.3	13 ± 2
11-fcc	19 ± 3	140 ± 20	4.6 ± 0.5	
12-fcc	11 ± 2	250 ± 30	2.1 ± 0.2	
13-fcc	12 ± 2	160 ± 20	2.6 ± 0.3	
14-bcc	12 ± 2	280 ± 30	n.a.	15 ± 2
14-fcc	28 ± 4	220 ± 30	1.1 ± 0.1	
15-bcc	20 ± 3	260 ± 30	n.a.	11 ± 2
16-hcp	11 ± 2	90 ± 10	n.a.	
17-fcc	15 ± 2	150 ± 20	1.9 ± 0.2	
18-fcc	7 ± 2	120 ± 20	3.8 ± 0.4	8 ± 2
19-fcc	18 ± 3	180 ± 20	4.6 ± 0.5	

**Table 3:** The hardness (H) and the elastic modulus (E) as obtained by nanoindentation. The error was calculated as two times the standard deviation of nine individual measurements.

No. of Position	H [GPa]	E [GPa]
1	8.4 ± 0.4	199 ± 8
2	10.0 ± 0.4	239 ± 5
3	9.3 ± 0.5	224 ± 5
4	9.4 ± 0.5	226 ± 6
5	8.5 ± 0.5	212 ± 7
6	11.0 ± 0.5	241 ± 6
7	10.3 ± 0.4	208 ± 5
8	10.6 ± 0.6	234 ± 6
9	11.8 ± 1.2	237 ± 12
10	9.6 ± 1.2	182 ± 14
11	10.2 ± 0.5	222 ± 7
12	11.1 ± 0.5	232 ± 6
13	10.5 ± 0.9	237 ± 10
14	8.5 ± 0.5	184 ± 5
15	9.2 ± 0.9	191 ± 8
16	10.9 ± 1.4	204 ± 11
17	9.6 ± 0.9	201 ± 9
18	10.4 ± 0.4	191 ± 5
19	10 ± 0.7	184 ± 9

**Table 4:** Comparison of the experimental and equilibrium phase compositions for the nineteen studied locations. For points with multiphase structures, the order of the phases reflects a descending volume fraction.

No. of Position	Experimental Phase Composition	Equilibrium Phase Composition
1	fcc	n.a.
2	fcc	n.a.
3	fcc	fcc + bcc
4	fcc	fcc + bcc
5	fcc	fcc + bcc
6	fcc	n.a.
7	fcc	fcc
8	fcc	fcc
9	hcp + fcc	hcp + bcc
10	fcc	n.a.
11	fcc	n.a.
12	fcc	fcc
13	fcc	n.a.
14	fcc + bcc	fcc + bcc
15	bcc	fcc + bcc
16	hcp + fcc	n.a.
17	fcc	fcc
18	fcc + hcp	n.a.
19	fcc	n.a.

## Data Availability

The evaluated data presented in this study are available in the tables of this paper. The raw measured data of this study are available on request from the corresponding author.
